# Analyzing large-scale samples confirms the association between rs16892766 polymorphism and colorectal cancer susceptibility

**DOI:** 10.1038/srep07957

**Published:** 2015-01-22

**Authors:** Mingzhi Liao, Guangyu Wang, Baoku Quan, Xingsi Qi, Zhihui Yu, Rennan Feng, Liangcai Zhang, Yongshuai Jiang, Yanqiao Zhang, Guiyou Liu

**Affiliations:** 1 College of Life Science, Northwest A&F University, Yangling, Shaanxi, China; 2 Department of Gastrointestinal Medical Oncology, The Affiliated Tumor Hospital of Harbin Medical University, Harbin, China; 3 Department of Oncology, The First Hospital of Harbin, Harbin, China; 4 Department of General Surgery, The First Hospital of Harbin, Harbin, China; 5 Department of Gastroenterology, The First Hospital of Harbin, Harbin, China; 6 Department of Gastroenterology, General Hospital of Heilongjiang Land Reclamation Bureau, Harbin, China; 7 Department of Nutrition and Food Hygiene, School of Public Health, Harbin Medical University, Harbin, China; 8 Department of Statistics, Rice University, Houston, TX, USA; 9 College of Bioinformatics Science and Technology, Harbin Medical University, Harbin, China; 10 Genome Analysis Laboratory, Tianjin Institute of Industrial Biotechnology, Chinese Academy of Sciences, China

## Abstract

Colorectal cancer (CRC) is a common complex disease caused by the combination of genetic variants and environmental factors. Genome-wide association studies (GWAS) have been performed and reported some novel CRC susceptibility variants. The rs16892766 (8q23.3) polymorphism was first identified to be significantly associated with CRC in European ancestry. The following studies investigated this association in Chinese, Japanese, Romanian, Swedish, African American, European American, and Croatian populations. These studies reported consistent and inconsistent results. Here, we reevaluated this association using the relatively large-scale samples from 13 studies (N = 59737, 26237 cases and 33500 controls) using a meta-analysis by searching the PubMed, Google Scholar and CRCgene databases. We observed no significant heterogeneity among the included studies. Our results showed significant association between rs16892766 polymorphism and CRC (*P* = 1.33E-35, OR = 1.23, 95% CI 1.20-1.27). Collectively, our analysis further supports previous findings that the rs16892766 polymorphism is significantly associated with CRC susceptibility. We believe that our findings will be very useful for future genetic studies on CRC.

Colorectal cancer (CRC), also called colon cancer or large bowel cancer, is the third most common form of cancer and the second leading cause of cancer-related death in the Western world and its lifetime risk in the United States is about 7%^1^. CRC is a common complex disease caused by the combination of genetic variants and environmental factors^1^. Genome-wide association studies (GWAS) are considered to be a new and power approach to detect the genetic variants of human complex diseases. Recently, GWAS have been performed and reported some novel CRC susceptibility variants^2^,^3^,^4^,^5^,^6^.

The rs16892766 (8q23.3) polymorphism was first identified to be significantly associated with CRC in European ancestry (*P* = 3.30E-18, the odds ratio (OR) = 1.25, 95% confidence interval (CI) 1.19-1.32, Minor allele = C)^6^. Based on the different genetic architecture, it is important to investigate whether rs16892766 polymorphism is associated with CRC risk in other ethnic populations. The following studies investigated this association in Chinese, Japanese, Romanian, Swedish, African American, European American, and Croatian populations^6^,^7^,^8^,^9^,^10^,^11^,^12^,^13^,^14^. The results showed that rs16892766 was not polymorphic in Chinese and Japanese populations^12^,^15^,^16^. The other studies reported consistent and inconsistent results for the association between rs16892766 and CRC. Some studies reported significant association between rs16892766 and CRC (*P* < 0.05)^6^,^8^,^9^,^13^,^14^, and the other studies reported no association between rs16892766 and CRC (*P* > = 0.05)^7^,^10^,^11^,^12^.

Recent studies investigated the influence of rs16892766 in Lynch syndrome. Wijnen et al. genotyped the rs16892766 polymorphism in 675 individuals from 127 different families from the Dutch Lynch syndrome Registry whose mutation carrier status was known^17^. They found a significant association between CRC risk and rs16892766 (8q23.3). The possession of the C-allele was associated with an elevated risk of CRC in a dose-dependent fashion, with homozygosity for CC being associated with a 2.16-fold increased risk^17^. Talseth-Palmer et al. investigated whether the rs16892766 (8q23.3) acts as modifier of disease risk in patients with Lynch syndrome using 684 mutation-positive patients with Lynch syndrome from 298 Australian and Polish families^18^. They identified an association between rs16892766 on chromosome 8q23.3 and the risk of developing CRC and age of diagnosis was found in MLH1 mutation carriers^18^.

It is reported that meta-analysis method involves combining and analyzing quantitative evidence from related studies to produce results based on a whole body of research^19^. It is a quantitative, formal, epidemiological study design used to systematically assess previous research studies to derive conclusions about that body of research^20^. The motivation of a meta-analysis is to aggregate information in order to achieve a higher statistical power. Considering the important role of rs16892766 polymorphism in CRC risk and inconsistent results reported by previous studies, we reevaluated this association using the relatively large-scale samples from 13 studies (N = 59737, 26237 cases and 33500 controls) using meta-analysis method by searching the PubMed, Google Scholar and CRCgene databases^21^.

## Methods

### Literature search

We searched the PubMed database to select all possible studies with key words including ‘rs16892766' and ‘colorectal cancer' or ‘8q23.3' and ‘colorectal cancer'. The literature search was updated on June 5, 2014. Meanwhile, we used the Google Scholar (http://scholar.google.com/) to query the articles citing the studies and all references in these studies identified by the PubMed. We selected only published articles written in English. Theodoratou et al. report the first comprehensive field synopsis and creation of a parallel publicly available and regularly updated database (CRCgene) that catalogs all genetic association studies on colorectal cancer (http://www.chs.med.ed.ac.uk/CRCgene/)^21^. They carried out meta-analyses to derive summary effect estimates for 92 polymorphisms in 64 different genes.

### Inclusion criteria

We selected the studies meeting the following criteria: (1) the study was conducted by a case-control design; (2) the study evaluated the association between rs16892766 polymorphism and CRC; (3) the study provided the numbers of rs16892766 genotypes or (4) the study must provided sufficient data to calculate the numbers of rs16892766 genotypes or (5) the study provided an OR with 95% CI as well as the *P* value; or (6) the study must provided sufficient data to calculate the OR and 95% CI;

### Data extraction

We extracted the following information from each study: (1) the name of the first author; (2) the year of publication; (3) the population and ethnicity; (4) the numbers of AD cases and controls; (5) the genotype numbers of rs16892766 polymorphism in cases and controls; (6) the numbers of rs16892766 genotypes or (7) to calculate the numbers of rs16892766 genotypes; (8) the OR with 95% CI or (9) to calculate the OR and 95% CI; All relevant calculations were completed using the program R (http://www.r-project.org/).

### Genetic model

The rs16892766 polymorphism has two alleles including C and A. C is the minor allele. We assume that C is the high-risk allele and A is the lower-risk allele. We selected the additive genetic model for further meta-analysis. The additive model can be described as C allele versus A allele^22^.

### Heterogeneity test

We evaluated the genetic heterogeneity among the studies included using Cochran's Q test, which approximately follows a *X*^2^ distribution with k-1 degrees of freedom (k stands for the number of studies for analysis). 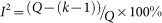, which ranges from 0 to 100%, was also used^23^. *I*^2^ is a measure of heterogeneity and a statistic that indicates the percentage of variance in a meta-analysis that is attributable to study heterogeneity^24^. Low, moderate, large and extreme heterogeneity corresponded to 0–25%, 25–50%, 50–75% and 75–100%, respectively^23^. The significant levels for heterogeneity are defied to be with *P* < 0.01 and *I*^2^ > 50%.

### Meta-analysis

If there is no significant heterogeneity among the included studies, the pooled OR is calculated by the fixed effect model (Mantel-Haenszel), otherwise the OR is calculated by random-effect model (DerSimonian-Laird). Z test is used to determine the significance of OR. All statistical tests for heterogeneity and meta-analysis were computed using R Package (http://cran.r-project.org/web/packages/meta/index.html).

### Sensitivity and publication bias analyses

We evaluated the relative influence of each study by omitting each study at a time. Meanwhile, we used funnel plots to evaluate the potential publication bias^25^. Begg and Egger's tests were used to evaluate the asymmetry of the funnel plot^25^.

## Results

### Literature search

**Figure 1:**
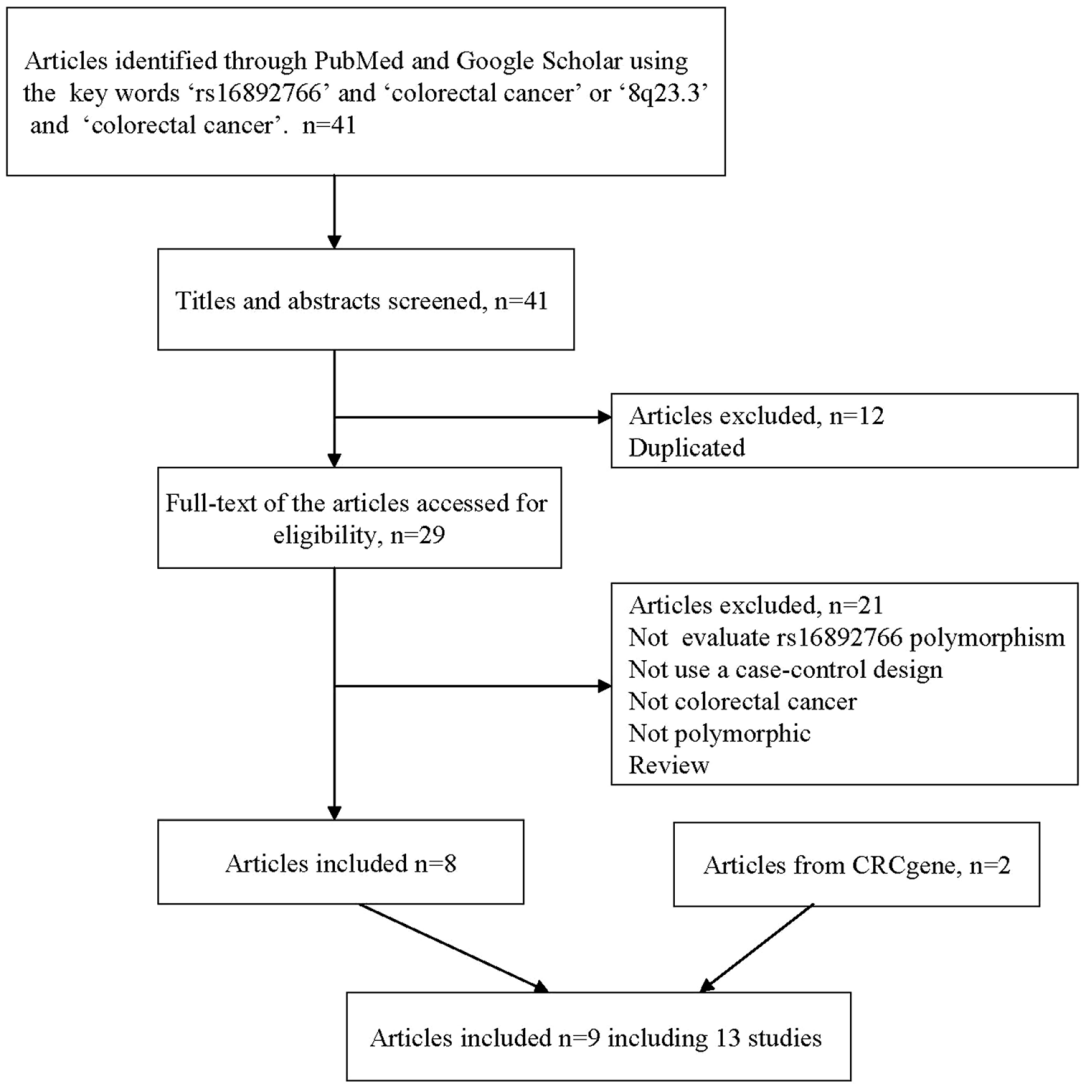
Flow chart of meta-analysis for exclusion or inclusion of individual articles. The selected studies must meet the following criteria: the study (1) was conducted by a case-control design; (2) evaluated the association between rs16892766 polymorphism and CRC; (3) provided the numbers of rs16892766 genotypes or (4) must provided sufficient data to calculate the numbers of rs16892766 genotypes or (5) provided an OR with 95% CI as well as the *P* value; or (6) must provided sufficient data to calculate the OR and 95% CI; OR, odds ratio; CI, confidence interval.

We selected 41 articles from PubMed and Google Scholar databases, and two articles from the CRCgene database. Finally, 9 articles including 13 independent studies were included for our following analysis. More detailed information about the inclusion or exclusion of selected studies was described in Figure 1. The main characteristics of the included studies are described in Table 1, which included the name of the first author, the year of publication, the population or ethnicity, the numbers of AD cases and controls, and the OR with 95% CI.

### Heterogeneity test

We evaluated the genetic heterogeneity of rs16892766 polymorphism among the selected studies using additive model and 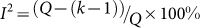 as well as *P* value. We did not identify significant heterogeneity among these 13 studies using additive model (*P* = 0.8239 and *I*^2^ = 0%).

### Meta-analysis

**Figure 2:**
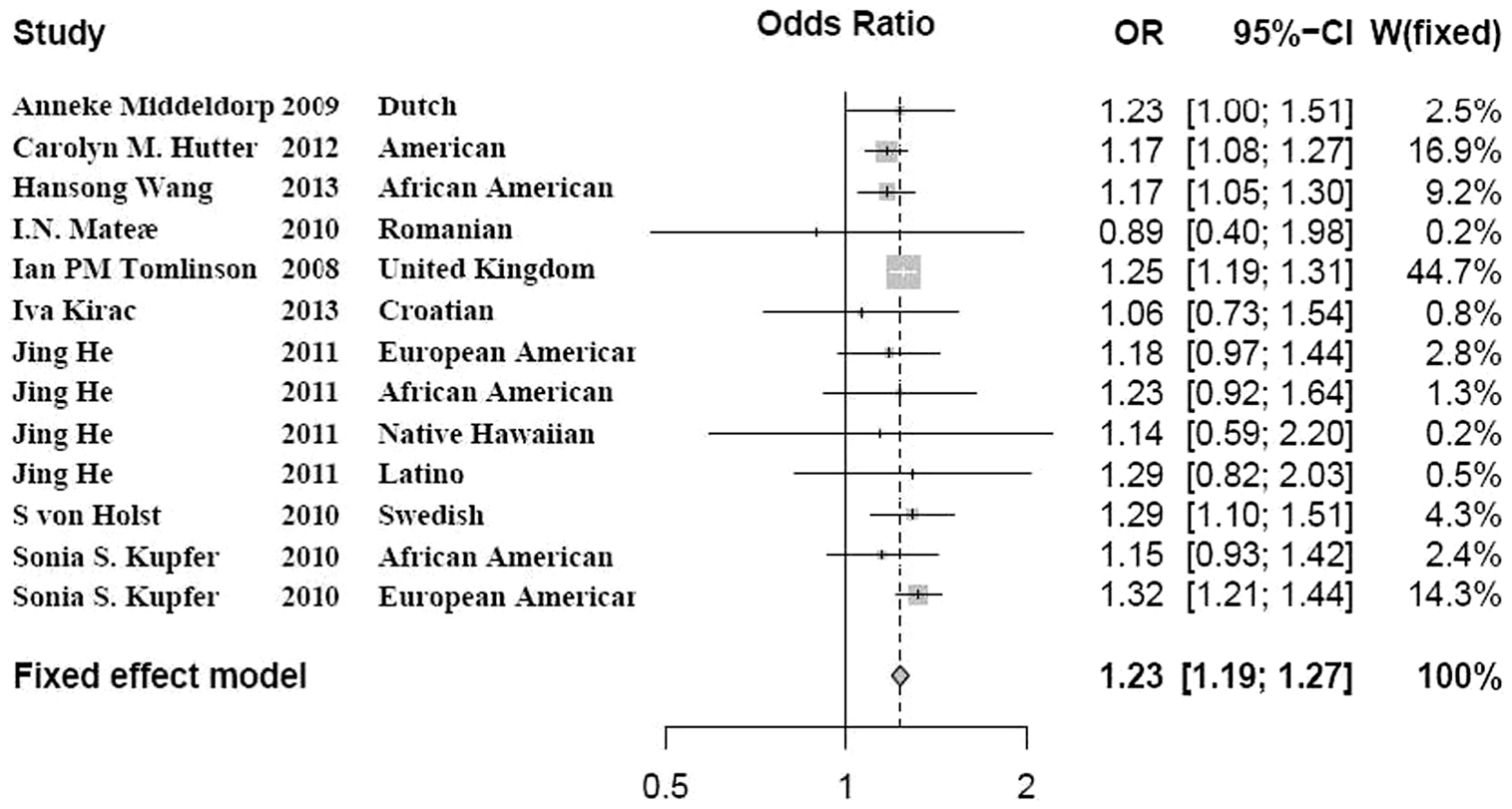
Forest plot for the meta-analysis of the rs16892766 polymorphism using additive model. 13 studies investigating rs16892766 polymorphism were included for meta-analysis. The heterogeneity among these 13 studies was evaluated by 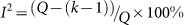 as well as *P* value. For each study, we list the name of the first author, the year of publication, the population or ethnicity, the OR with 95% CI and the weight in meta-analysis. For the meta-analysis, the overall OR was calculated by the fixed effect model. OR, odds ratio; CI, confidence interval; fixed, fixed effect model.

As described above, we identified no significant heterogeneity among these 13 studies. We then performed a meta-analysis. We calculated the overall OR by the fixed effect model. Our results showed significant association between rs16892766 polymorphism and CRC using additive model (*P* = 1.33E-35, OR = 1.23, 95% CI 1.20-1.27). In Figure 2, for each study, we list the name of the first author, the year of publication, the population or ethnicity, the OR with 95% CI and the weight in meta-analysis. Detailed results are described in Figure 2.

### Sensitivity analysis and publication bias analysis

**Figure 3:**
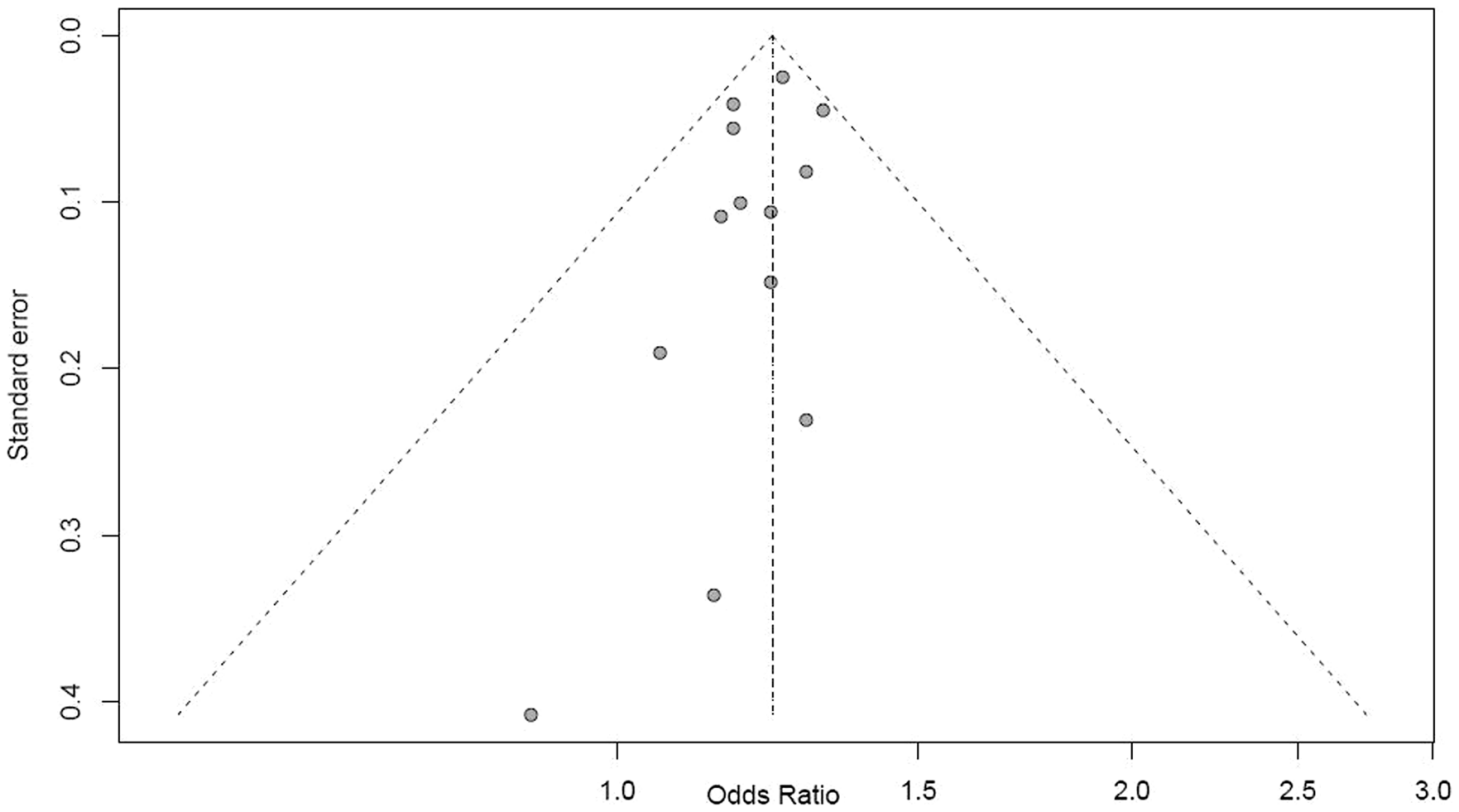
Funnel plot for publication bias analysis of rs16892766 polymorphism in CRC using additive model. This funnel plot is based on the 13 studies investigating rs16892766 polymorphism in meta-analysis. The X-axis stands for the ORs and the Y-axis is the standard error for each of the 13 studies. Begg and Egger's tests were used to evaluate the asymmetry of the funnel plot.

By excluding any one study, we identified that the association between rs16892766 polymorphism and CRC did not vary substantially. The funnel plots are symmetrical inverted funnels for models (Figure 3), which suggest no significant publication bias for the additive model (Begg's test, *P* = 0.2206 and Egger's test, *P* = 0.2206).

## Discussion

Recent GWAS identified rs16892766 (8q23.3) polymorphism to be significantly associated with CRC in European ancestry^6^. The following studies investigated this association and reported consistent and inconsistent results. It is important to assess the genetic architecture of rs999737 polymorphism across different populations. Here, we reevaluated this association using the relatively large-scale samples from 13 studies by searching the PubMed, Google Scholar and CRCgene databases. We first evaluated the genetic heterogeneity of rs16892766 polymorphism among the selected studies. We did not identify significant heterogeneity among these 13 studies using additive model (*P* = 0.8239 and *I*^2^ = 0%). We then conducted a meta-analysis using fixed effect model. Our results showed significant association between rs16892766 polymorphism and CRC using additive model (*P* = 1.33E-35, OR = 1.23, 95% CI 1.20-1.27). Collectively, our analysis further supports previous findings that the rs16892766 polymorphism is significantly associated with CRC susceptibility. We believe that our findings will be very useful for future genetic studies on CRC.

Before our submission, we accessed the PubMed and Google Scholar databases using the key words ‘rs16892766' and ‘meta'. We identified two articles^8^,^21^. Hutter et al. examined potential effect-modification between 10 loci and probable or established environmental risk factors for CRC in 7,016 CRC cases and 9,723 controls from nine cohort and case-control studies^8^. They used meta-analysis of an efficient empirical-Bayes estimator to detect potential multiplicative interactions between each of the SNPs and select major CRC risk factors^8^. The strongest statistical evidence for a gene-environment interaction across studies was for vegetable consumption and rs16892766, located on chromosome 8q23.3, near the EIF3H and UTP23 genes^8^. Theodoratou et al. carried out meta-analyses to derive summary effect estimates for 92 polymorphisms in 64 different genes and constructed the CRCgene database (http://www.chs.med.ed.ac.uk/CRCgene/)^21^.

Our study is different from previous studies^8^,^21^. Hutter et al. investigated the gene-environment interaction between each of the SNPs and select major CRC risk factors^8^. We accessed CRCgene databases and found two articles including three studies investigating rs16892766 polymorphism. Here, we conducted an updated analysis to reevaluate the association between rs16892766 polymorphism and CRC using the relatively large-scale samples by searching the PubMed and Google Scholar databases. We observed no significant heterogeneity among the included studies. Our results from this meta-analysis are consistent with the findings from CRC GWAS. Our results showed association between rs16892766 polymorphism and CRC (*P* = 1.33E-35, OR = 1.23, 95% CI 1.20-1.27), which is more significant than previous GWAS (*P* = 3.30E-18, OR = 1.25, 95% CI 1.19-1.32)^6^.

Pittman et al. generated a fine scale map of a 300 Kb region encompassing the rs16892766 association signal using 1,964 CRC cases and 2,081 controls^26^. A 22 kb genomic region of linkage disequlibrium (LD; Chr8:117,690,773–117,712,909) capturing rs16892766 provided the best evidence for the 8q23 CRC association signal^26^. Four most significantly associated SNPs-rs16892766, Novel 28, rs16888589 and rs11986063 are strongly correlated with one another (pairwise r2 > 0.75) and constitute a single risk haplotype^26^. Reporter gene studies demonstrated that the rs16888589, which was in high LD with rs16892766, acts as an allele-specific transcriptional repressor^26^. Chromosome conformation capture analysis showed that the genomic region harboring rs16888589 interacts with the promoter of gene for eukaryotic translation initiation factor 3, subunit H (EIF3H)^26^. EIF3H is located at 8q23 and identified to be a CRC susceptibility gene by previous GWAS^6^,^27^. Increased expression of EIF3H gene increases CRC growth and invasiveness thereby providing a biological mechanism for the 8q23.3 association^26^.

Despite these interesting results, our study has a limitation. Here, we investigated the association between rs16892766 and CRC using additive model. It is reported that most meta-analyses used an additive genetic model^28^. In general, this model performs well when the true underlying genetic model is uncertain^28^. It was also important to analyze the association between rs16892766 and CRC using dominant model (CC+CA versus AA) and recessive model (CC versus CA+AA)^22^. Exact genotype numbers of all studies used in our analysis are required for the dominant and recessive models. We attempted to obtain these genotype numbers but were not successful. Considering that the original genotype data are not publicly available for us, future replication studies using genotype data are required to replicate our findings.

## Author Contributions

G.Y.L., Y.S.J. and M.Z.L. conceived and initiated the project, searched the PubMed database and extracted the information from each study. G.Y.L., B.K.Q., X.S.Q., Z.H.Y. and G.Y.W. analyzed the data. R.N.F. and L.C.Z. prepared the figures 1–3. G.Y.L., Y.S.J., M.Z.L. and Y.Q.Z. wrote the manuscript. All authors reviewed the manuscript, and contributed to the final manuscript.

## Figures and Tables

**Table 1:** Main characteristics of the included studies investigating the association between rs16892766 and colorectal cancer

Study	Year	Population or Ethnicity	Case #	Control #	OR	CI (Down)	CI (Up)
Anneke Middeldorp^7^	2009	Dutch	995	1340	1.23	1	1.5
Carolyn M. Hutter^8^	2012	American, Canada and Europe	7016	9723	1.17	1.08	1.27
Hansong Wang^9^	2013	African American	1894	4703	1.17	1.05	1.32
I.N. Mateæ^10^	2010	Romanian	92	96	0.89	0.4	1.97
Ian PM Tomlinson^6^	2008	United Kingdom	10,731	10,961	1.25	1.19	1.32
Iva Kirac^11^	2013	Croatian	291	594	1.06	0.73	1.54
Jing He^12^	2011	European American	1171	1534	1.18	0.97	1.43
Jing He^12^	2011	African American	382	510	1.23	0.92	1.63
Jing He^12^	2011	Native Hawaiian	323	472	1.14	0.59	2.21
Jing He^12^	2011	Latino	393	524	1.29	0.82	2.05
S von Holst^13^	2010	Swedish	1755	1691	1.29	1.1	1.51
Sonia S Kupfer^14^	2010	African American	795	985	1.15	0.93	1.41
Sonia S Kupfer^14^	2010	European American	399	367	1.32	1.21	1.44
N = 59737			N = 26237	N = 33500			
